# Sex differences in the evaluation of proteinuria using the urine dipstick test

**DOI:** 10.3389/fmed.2023.1148698

**Published:** 2023-06-26

**Authors:** Chiari Kojima, Hiroshi Umemura, Tatsuo Shimosawa, Tomohiro Nakayama

**Affiliations:** ^1^Department of Clinical Laboratory, International University of Health and Welfare Mita Hospital, Tokyo, Japan; ^2^Division of Laboratory Medicine, Department of Pathology and Microbiology, Nihon University School of Medicine, Tokyo, Japan; ^3^Department of Clinical Laboratory, School of Medicine, International University of Health and Welfare, Chiba, Japan

**Keywords:** urine dipstick test, urine protein creatinine ratio, trace proteinuria, proteinuria, urine specific gravity

## Abstract

**Background:**

The urine protein dipstick test is widely used, but false-positive and false-negative results may occur. This study aimed to compare the urine protein dipstick test with a urine protein quantification method.

**Methods:**

The data were extracted using the Abbott Diagnostic Support System, which analyzes the inspection results using multiple parameters. This study included 41,058 specimens tested using the urine dipstick test and protein creatinine ratio from patients aged ≥18 years. The proteinuria creatinine ratio was classified according to the Kidney Disease Outcomes Quality Initiative guidelines.

**Results:**

Urine protein on the dipstick test was negative in 15,548 samples (37.9%), trace in 6,422 samples (15.6%), and ≥1+ in 19,088 samples (46.5%). Among the trace proteinuria samples, A1 (<0.15 g/gCr), A2 (0.15–0.49 g/gCr), and A3 (≥0.5 g/gCr) category proteinuria accounted for 31.2, 44.8, and 24.0% of samples, respectively. All trace proteinuria specimens with a specific gravity of <1.010 were classified as A2 and A3 category proteinuria. In the trace proteinuria cases, women had a lower specific gravity and a higher percentage of A2 or A3 category proteinuria than men. The sensitivity in the “dipstick proteinuria” ≥ trace” group was higher than that in the “dipstick proteinuria ≥ 1+” group within the lower specific gravity group. The sensitivity in the “dipstick proteinuria ≥ 1+” group was higher for men than for women, and the sensitivity in the “dipstick proteinuria ≥ trace” group was higher than that in the “dipstick proteinuria ≥ 1+” group for women.

**Conclusion:**

Pathological proteinuria assessment requires caution; this study suggests that evaluating the specific gravity of urine specimens with trace proteinuria is essential. Particularly for women, the sensitivity of the urine dipstick test is low, and caution is needed even with trace specimens.

## 1. Introduction

Proteinuria is an independent risk factor for end-stage renal disease (ESRD), cardiovascular disease, and decreased lifespan. Dipstick proteinuria can predict the incidence of ESRD, myocardial infarction, and mortality (1–4). Chronic kidney disease (CKD) is often asymptomatic until advanced stages; hence, accurate evaluation of proteinuria and creatinine levels is essential for diagnosing and managing CKD.

The urine dipstick test is a convenient and inexpensive test widely used to screen for proteinuria during public health check-ups. The urine protein dipstick test preferentially detects albumin. There are several causes of false-positive or false-negative proteinuria results with a urinary dipstick test. False positives occur with dehydration, exercise, infection, and alkaline urine, while false negatives occur with dilute urine and non-albumin proteins, such as the Bence-Jones protein.

Several reports have highlighted the importance of trace proteinuria. It is widely accepted that trace proteinuria is a risk factor for all-cause and cardiovascular mortality (5–7). Any trace proteinuria detected by the urine dipstick test indicates a risk of metabolic syndrome, hypertension, or diabetes despite a normal estimated glomerular filtration rate (eGFR) (8). Growing evidence suggests that trace proteinuria is associated with a higher risk of developing atrial fibrillation (9) and heart failure (10). Non-dialysis CKD patients have an increased risk of cancer (11), and trace proteinuria may be a risk factor for cancer mortality (12).

As evidence of the prevalence and importance of CKD accumulates, assessment of asymptomatic proteinuria is becoming more critical than ever. Although trace proteinuria cannot be underestimated, it plays a minor role in general practice and health check-ups. In this study, the Diagnostic Support System (DSS), developed by Abbott Japan LLC, supports clinical diagnoses and reduces clinical errors by providing an algorithm-based analysis of test results.

Twenty-four hours of urine collection is considered the gold standard for quantifying proteinuria. Urine protein/creatinine ratio (uPCR) measurement can replace 24 h urine collection to quantify clinical proteinuria and is widely used in routine practice (13). Microalbuminuria is similarly calculated using the urine albumin/creatinine ratio (uACR); however, uACR measurement is limited in Japan to patients with diabetes. The evaluation of uPCR or uACR is also essential from the perspective of CKD severity assessment (14).

The accuracy of the urine dipstick test in detecting uACR (15–18) or uPCR (19) has been evaluated in several studies. However, most studies on urine dipstick test methods are based on health check-ups and community-based studies, and few studies have been conducted on hospital-visited patients. The aim of this study was to explore the differences between urine dipstick test results and uPCR to investigate the problems with urinalysis evaluation. Furthermore, we aimed to find ways to improve the accuracy of diagnosis using factors such as age, sex, and eGFR values using the Abbott DSS.

## 2. Materials and methods

### 2.1. Study population

We included both inpatients and outpatients aged ≥18 years who underwent urinalysis at our institution in Tokyo, Japan, between January 2018 and August 2020. Our study focused on patients whose dipstick and uPCR tests were conducted on the same day. A total of 41,058 urine specimens were compared to the urine dipstick test and uPCR performed on the same specimen. Some samples were obtained from the same patient at different time points.

### 2.2. Urine and blood tests

Urinalysis was performed using a dipstick in a fresh urine specimen (UA test 1,000 AD; Techno Medica Co.). Urine protein was measured using a pyrogallol red absorptiometric method (Micro TP-AR2; Fujifilm Wako Pure Chemical Co.), and urine creatinine was measured using an enzymatic method (Determiner L CRE; Minaris). The uPCR was calculated by dividing the urinary protein concentration by the urinary creatinine concentration. The uPCR was stratified according to the Kidney Disease Outcomes Quality Initiative guidelines (A1: <0.15 g/gCr, A2: 0.15–0.49 g/gCr, A3: ≥0.5 g/gCr) (14).

The serum creatinine levels were measured using an enzymatic method. The eGFR was calculated using the Japanese Nephrology Society equation (20).

### 2.3. Abbott diagnostic support system

**Figure 1:**
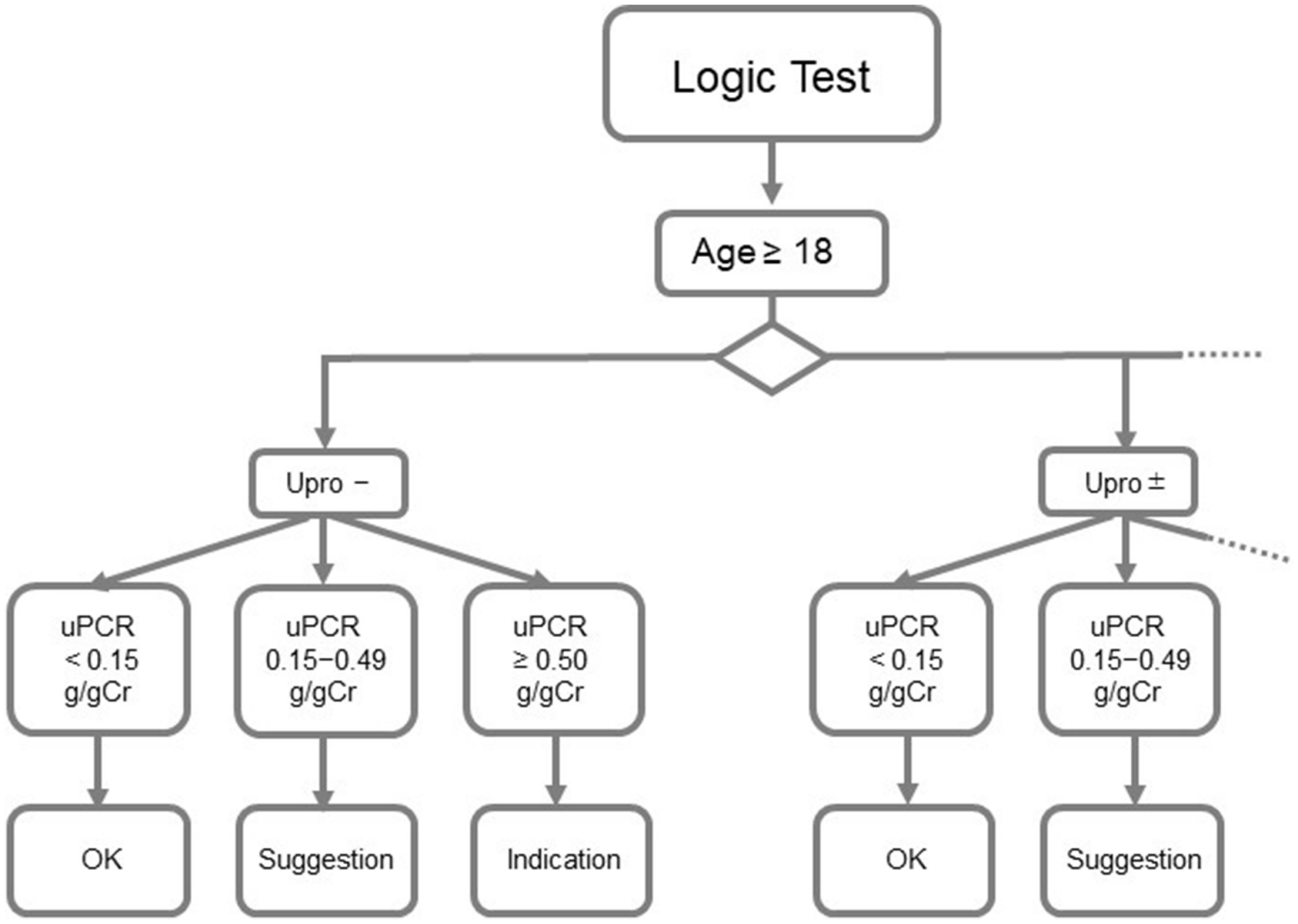
Abbott diagnostics support system. Upro, urinary protein; uPCR, urinary protein/creatinine ratio.

The logic for analysis was designed using clinical parameters, such as age, sex, urine test paper results, and urine quantification results (Figure 1). Each result is categorized as “OK,” “suggestion,” or “indication.” The test results received from CLNILAN GL-3 (A&T Co.), a clinical laboratory system, from January 2018 to August 2020, were automatically analyzed by the DSS based on the logic for analysis.

### 2.4. Statistical analyses

Data analysis was performed using GraphPad Prism (version 9.4.0; San Diego, CA, United States). The Pearson’s chi-squared test was used to assess the association between dipstick proteinuria and uPCR. In addition, we calculated the sensitivity, specificity, positive predictive value, and negative predictive value of the trace dipstick protein for detecting A3 category proteinuria.

### 2.5. Ethical statement

This study was approved by the Institutional Review Board of Nihon University Itabashi Hospital (RK-190212-5). All procedures in this study were performed according to the ethical standards of the Nihon University Itabashi Hospital and complied with the Declaration of Helsinki of the World Medical Association.

## 3. Results

### 3.1. Urinalysis results and clinical background

The clinical characteristics of the patients enrolled in the study are shown in Table 1. Approximately half of the samples were in the urinary protein A3 category, and eGFR stages were better than G3a. Of the total samples, the urinary protein A1 category accounted for 33.1%, the urinary protein A2 category for 24.0%, and the urinary protein A3 category for 42.9%. A total of 57.5% of the samples came from men (Table 1).

**Table 1:** Clinical characteristics of patients.

	Overall
Variables	*n* = 41,058
Dipstick proteinuria	
(−)	15,548 (37.9%)
(±)	6,422 (15.6%)
(1+)	8,470 (20.6%)
(2+)	8,340 (20.3%)
(3+)	2,031 (4.9%)
(4+)	247 (0.6%)
Urine protein/creatinine ratio	
<0.15 g/gCr	13,595 (33.1%)
0.15–0.49 g/gCr	9,866 (24.0%)
≥0.50 g/gCr	17,597 (42.9%)
Urinary occult blood	
(−)	24,525 (59.7%)
(±)	7,016 (17.1%)
(1+)	2,943 (7.2%)
(2+)	4,265 (10.4%)
≥(3+)	2,309 (5.6%)
Age category	
18–49 years	11,488 (27.9%)
50–69 years	4,714 (32.6%)
≥70 years	5,781 (39.4%)
Sex	
Man	23,588 (57.5%)
Woman	17,470 (42.5%)
eGFR stage (Overall:38,861)	
G1	3,654 (9.4%)
G2	9,701 (24.9%)
G3a	6,775 (17.4%)
G3b	7,546 (19.4%)
G4	6,941 (17.8%)
G5	4,244 (10.9%)

eGFR, estimated glomerular filtration rate.

### 3.2. Comparison of dipstick protein level with urine protein/creatinine ratio

The percentages of A1, A2, and A3 category proteinuria for each dipstick protein level are shown in Table 2. The uPCR levels tended to correspond with semi-quantitative proteinuria according to the urine dipstick test.

**Table 2:** Comparison of urine dipstick test results and urine protein category results.

		Urine protein/creatinine ratio						Total
		<0.15 g/Cr		0.15–0.49 g/Cr		≥0.50 g/Cr		
		*n*	%	*n*	%	*n*	%	
Dipstick proteinuria	−	11,039	71.0%	4,007	25.8%	502	3.2%	15,548
	Trace	2,005	31.2%	2,874	44.8%	1,543	24.0%	6,422
	1+	510	6.0%	2,526	29.8%	5,434	64.2%	8,470
	2+	40	0.5%	436	5.2%	7,864	94.3%	8,340
	3+	1	0.1%	23	1.1%	2,007	98.8%	2,031
	4+	0	0.0%	0	0.0%	247	100%	247

### 3.3. The impact of urine specific gravity on urine protein/creatinine ratio in trace proteinuria samples

**Figure 2:**
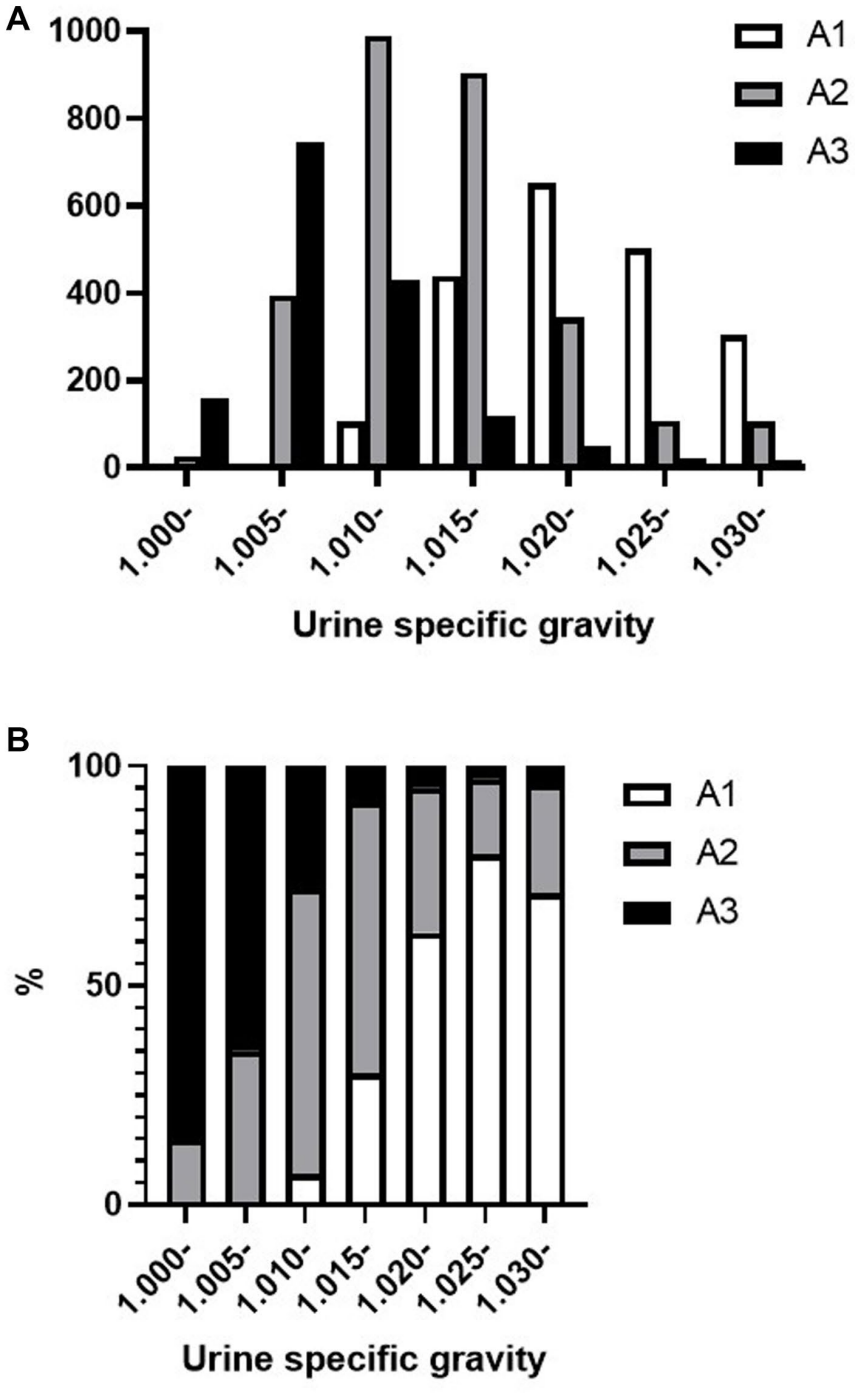
Distribution of urine specific gravity and A1–A3 category proteinuria in trace proteinuria samples. **(A)** Total number of A1, A2, and A3 category proteinuria and **(B)** A1, A2, and A3 category proteinuria occupancy for each specific gravity.

Figures 2A,B shows the distribution of the urine specific gravity (USG) and proteinuria A1–A3 categories among the trace proteinuria samples tested using the urine dipstick test. The dipstick category of trace specimens in the A3 category had USG distributed under 1.015, whereas in the A2 category, the USG was widely distributed. Of the trace samples with USG < 1.010, two samples (0.2%) were in the A1 category, 422 samples (31.7%) were in the A2 category, and 906 samples (68.1%) were in the A3 category.

### 3.4. Differences in age, sex, and glomerular filtration rates in A3 category proteinuria with trace proteinuria samples

**Figure 3:**
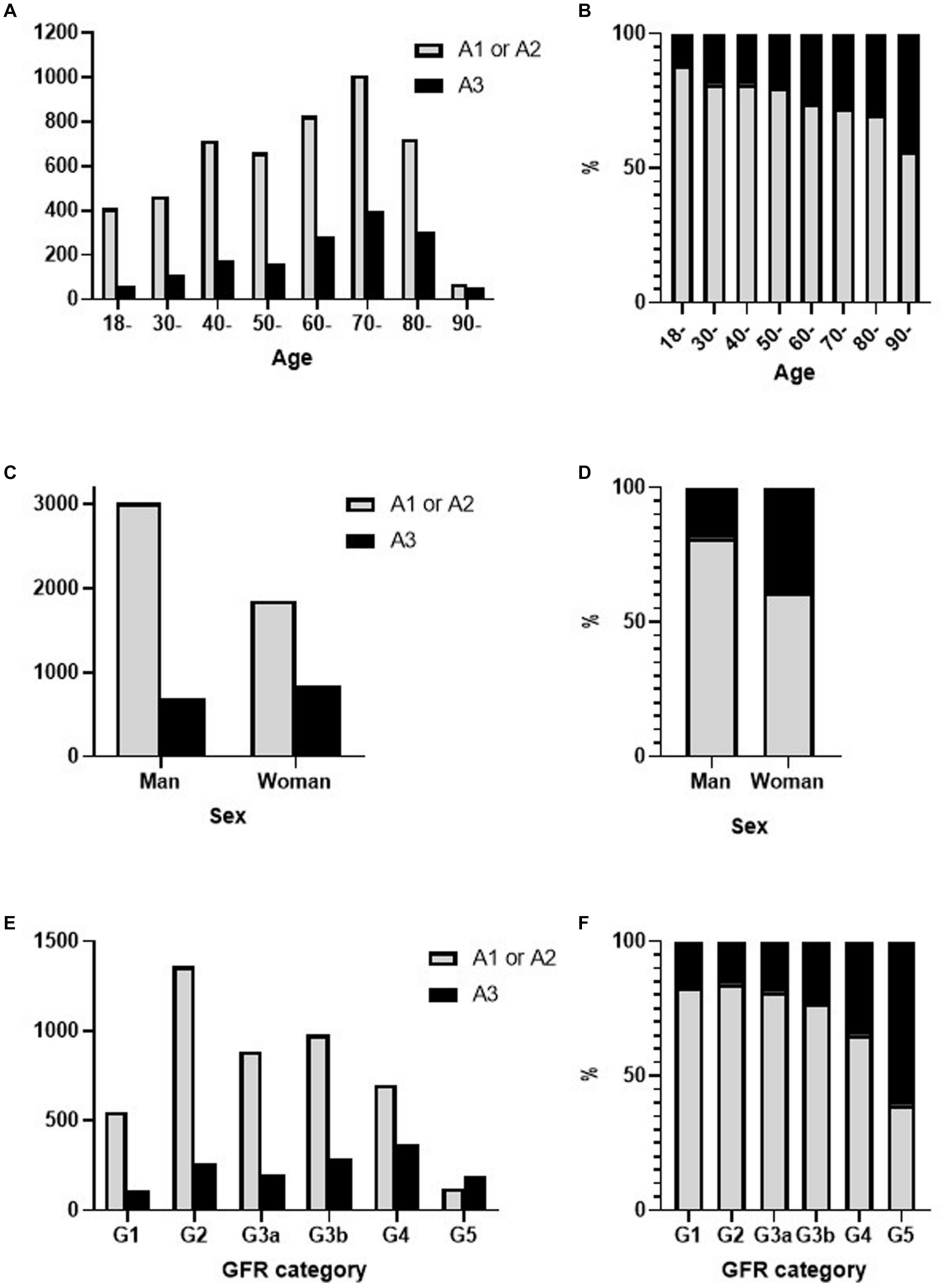
Frequency differences in **(A,B)** age, **(C,D)** sex, and **(E,F)** eGFR values in uPCR A1–A2 or A3 categories with trace proteinuria. eGFR, estimated glomerular filtration rate; uPCR, urinary protein/creatinine ratio.

A3 category proteinuria with trace proteinuria using the urine dipstick test was more prevalent in the older population (Figures 3A,B). Among the trace proteinuria samples, the A3 category proteinuria accounted for 18.8% of the samples from men and 31.2% of the samples from women, indicating that women accounted for a higher percentage (Figures 3C,D). The frequency of proteinuria in the A3 category increased with lower eGFR (Figures 3E,F).

### 3.5. Differences between urine dipstick test results and uPCR results by sex

**Figure 4:**
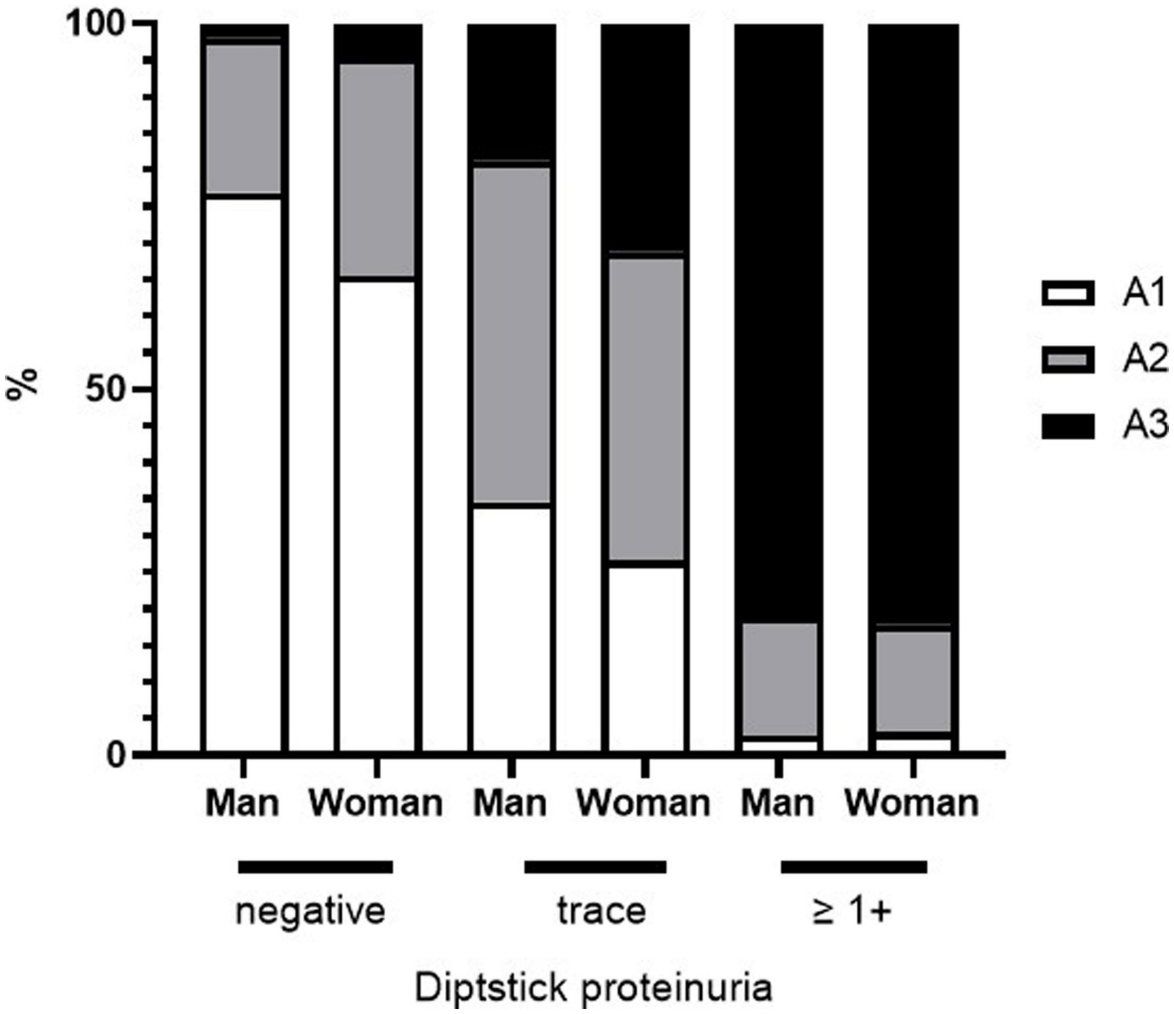
Sex differences in the distribution of A1–A3 category proteinuria and urinary protein dipstick values.

**Figure 5:**
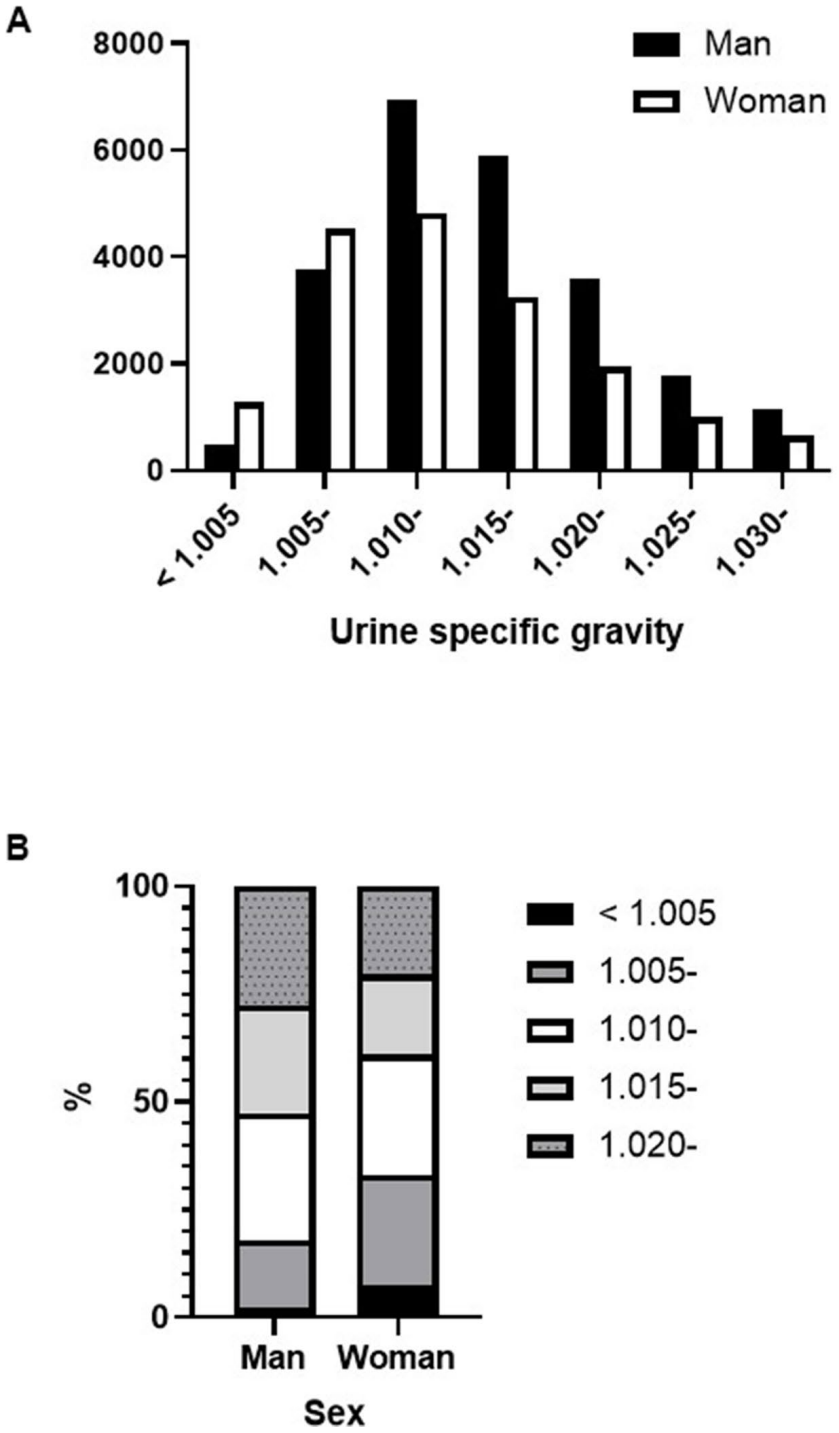
Differences in urine specific gravity between sexes. **(A)** Number of specimens for each urine specific gravity level by sex. **(B)** Differences in urine specific gravity distribution by sex.

Figure 4 shows the percentage of proteinuria in the A1, A2, and A3 categories according to sex and dipstick test results. For negative or trace dipstick tests, sex influenced the discrepancy between the dipstick and uPCR test results. A3 category proteinuria with trace positive results was more abundant in women than in men (Figure 4). The same sex-related differences were found in USG. A greater significant proportion of women had lower USG samples than men (Figures 5A,B). In contrast, there were no sex differences in the CKD eGFR category or age (data not shown).

### 3.6. Diagnostic accuracy of urine protein dipstick test for the detection of proteinuria A3 category

Table 3 shows the diagnostic accuracy of the urine protein dipstick test with trace or higher for detecting A3 category proteinuria. We examined the effect of USG on test accuracy based on either 1.010 or 1.015, which were the modes of the urine specimens. Among lower specific gravity groups, the sensitivity was lower for the “dipstick proteinuria ≥ 1+” group compared to the “dipstick proteinuria ≥ trace” group. In contrast, among higher specific gravity groups, the specificity was lower for the “dipstick proteinuria ≥ 1+” group than the “dipstick proteinuria ≥ trace” group. The sensitivity and specificity of the urine dipstick test, when trace or 1+ were examined by sex, showed that the sensitivity was higher in men. In the case of women, the sensitivity of the urine protein dipstick test to detect A3 category proteinuria was found to be higher for a positive result of “dipstick proteinuria ≥ trace” than a positive result of “dipstick proteinuria ≥ 1+.”

**Table 3:** Diagnostic accuracy of urine dipstick test results for detection of A3 category proteinuria.

	Dipstick ≥ trace for A3 proteinuria detection				Dipstick ≥ 1+ for A3 proteinuria detection			
	Sensitivity	Specificity	PPV	NPV	Sensitivity	Specificity	PPV	NPV
Total population	0.971	0.641	0.670	0.968	0.884	0.849	0.815	0.907
Man	0.986	0.580	0.666	0.980	0.922	0.817	0.811	0.924
Woman	0.948	0.715	0.677	0.956	0.823	0.888	0.822	0.889
Urine specific gravity <1.010	0.911	0.915	0.891	0.931	0.702	0.989	0.980	0.814
Urine specific gravity <1.015	0.956	0.819	0.833	0.952	0.830	0.954	0.945	0.856
Urine specific gravity ≥1.010	0.991	0.553	0.624	0.988	0.943	0.804	0.782	0.950
Urine specific gravity ≥1.015	0.995	0.479	0.522	0.994	0.965	0.753	0.691	0.974

PPV, positive predictive value; NPV, negative predictive value.

## 4. Discussion

In this study, two-thirds of trace proteinuria specimens using the urine dipstick test resulted in uPCR in the A2 or A3 category. In previous reports, zero to 5.8% of trace specimens by urine dipstick tests were in the A3 category and 7.1 to 17.2% in the A2 category (19, 21). Our study found that one-fourth of trace proteinuria specimens presented with A3-classified proteinuria. This was a more significant proportion than the previously reported health check-up subjects. This result suggests that CKD is more common in patients who are regularly seen at tertiary medical centers, that dipstick test screening for proteinuria has low sensitivity, and that quantitative urinary protein measurement is required.

USG has long been considered a useful surrogate marker of urine osmolality (22). It has been proposed that low USG results in poor detection of proteinuria in urine dipstick tests (23). Almost all low USG urine specimens (<1.010) in this study showed A2 or A3 category proteinuria. The sensitivity to detect A3 category proteinuria with urinary protein 1+ or higher was reduced with low USG. Our study suggests that more attention is needed to evaluate low USG samples. In addition to USG, our study confirmed that eGFR and age affected the accuracy of the urine dipstick test, as previously reported (19). The high incidence of false negatives in subjects with decreased eGFR or aging may be due to decreased urinary concentrating ability. Consistent with previous reports (24, 25), samples from older patients or those with low eGFR had a higher proportion of low USG in this study (data not shown).

The proteinuria detection accuracy of the urine dipstick test had low sensitivity in women, which is consistent with results that were reported when comparing the urine dipstick test with uPCR or uACR (15, 18, 19, 26). Interestingly, women constituted a higher percentage of patients with low USG in our study. Sex differences in urine concentration due to age, sodium intake, and level of kidney disease have also been reported (27). There are sex differences in the thirst threshold, arginine vasopressin (AVP) levels, and other regulatory mediators that may contribute to higher urine osmolality in men (27–29). Low USG in women, even before puberty (30) and after age 50 years, is considered postmenopausal (27), suggesting that sex hormones may not be responsible for the lower USG. In fact, the percentage of women with USG < 1.010 did not differ between the age groups older and younger than 50 years (data not shown).

The influence of pH should be considered when evaluating urinary protein levels using a urine dipstick test. False-positive results appear with alkaline urine (pH ≥ 8.0), and false-negative results occur with acidic urine (31). Indeed, alkaline urine and A3-classified proteinuria specimens frequently showed false-positive urine protein levels of 1+ or higher (data not shown). However, there was no increase in false negatives at low pH (pH < 5.0). Alkaline urine is more prevalent in women (32), and our study of trace proteinuria specimens showed that alkaline urine in women is more likely to have A3 category proteinuria (Supplementary Figure S1). In the evaluation of proteinuria using urine dipstick tests, false-positive results for alkaline urine should be noted.

The results of this study should be interpreted in light of several limitations. This study was conducted among patients in a university hospital, representing data from a broad patient population, including those with acute kidney injury (AKI), severe infection, cardiac disease, and pregnancy. Furthermore, the correlation between individual patient status and proteinuria was not analyzed in detail. Our study was conducted at a single center, and only one reagent was tested for proteinuria. The differences in results from various reagents have not been thoroughly investigated. Our analysis included only specimens for which both dipstick proteinuria and uPCR were requested. As a result, selection bias may have occurred during patient selection.

This study extracted laboratory data using a web-based clinical laboratory information system. Patient background information, other than age and sex, such as height, weight, comorbidities, and medication use, was not considered. Unfortunately, for the characteristics of the Abbott DSS, only stratified data could be compared, and numerical comparisons were not available.

We used uPCR for the quantitative evaluation of proteinuria. Since uPCR is calculated using urine creatinine concentrations, 24 h proteinuria may not be accurately evaluated in cases with low muscle mass or renal function. uPCR may also be overestimated in cases of low USG and underestimated it in cases of high USG (33). Some specimens may contain uPCR results that do not reflect 24 h proteinuria. The urine dipstick method can give false-negative results for Bence-Jones Protein; however, the presence of Bence-Jones Protein was not assessed in this study. Furthermore, the influence of macrohematuria, menstruation, and urinary tract infection was not considered.

The assessment of renal function by eGFR calculated from serum creatinine may not represent the actual renal function in patients with muscle disease or lower limb amputation. In patients with AKI, eGFR values are imprecise because of the large variability in creatinine levels. The formulas for calculating eGFR used in this study are for Japanese patients and may not be appropriate for patients of other ethnicities.

In this study, the influence of low specific gravity, which was frequent in samples from women, was a prominent cause of the false-negative results. Therefore, negative or trace proteinuria in women should be considered possible pathological proteinuria. In urine dipstick tests, alerts from the Abbott DSS regarding the addition of uPCR testing would be informative in evaluating and managing CKD. Particularly in samples with low USG or from women with trace urine protein, additional uPCR measurements would be beneficial.

## Data availability statement

The raw data supporting the conclusions of this article will be made available by the authors, without undue reservation.

## Ethics statement

The studies involving human participants were reviewed and approved by Nihon University Itabashi Hospital, Clinical Research Judging Committee. Written informed consent for participation was not required for this study in accordance with the national legislation and the institutional requirements.

## Author contributions

CK analyzed the data and drafted the article. HU and TN contributed to the conception and design of the study. TS and TN revised the article critically for important intellectual content. All authors contributed to the article and approved the submitted version.

## Funding

We received an annual donation of 300,000 yen from Abbott Japan. The funder was not involved in the study design, collection, analysis, interpretation of data, the writing of this article, or the decision to submit it for publication.

## Conflict of interest

The authors declare that the research was conducted in the absence of any commercial or financial relationships that could be construed as a potential conflict of interest.

## Publisher’s note

All claims expressed in this article are solely those of the authors and do not necessarily represent those of their affiliated organizations, or those of the publisher, the editors and the reviewers. Any product that may be evaluated in this article, or claim that may be made by its manufacturer, is not guaranteed or endorsed by the publisher.
