# Supplementary material for: Sex differences in the evaluation of proteinuria using the urine dipstick test
Source: Front Med (Lausanne). 2023 Jun 26;10:1148698. doi: 10.3389/fmed.2023.1148698 (PMC10332457; doi:10.3389/fmed.2023.1148698)
Supplement: Supplementary file 1 [file Data_Sheet_1.docx]

Supplementary Material

**Sex Differences in the Evaluation of Proteinuria Using the Urine Dipstick Test**

**Chiari Kojima, Hiroshi Umemura, Tatsuo Shimosawa, Nakayama Tomohiro^*^**

*** Correspondence:** Tomohiro Nakayama MD, PhD: nakayama.tomohiro@nihon-u.ac.jp

**Supplementary Figure 1.** Sex differences of urinary pH distribution (A,C) and proportion (B,D).

A

C

B

D
